# Diabetes Mellitus Manifests As Focal Dystonia

**DOI:** 10.7759/cureus.19899

**Published:** 2021-11-25

**Authors:** Hussam R Alkaissi, Essam Al-Sibahee

**Affiliations:** 1 Internal Medicine, Kings County Hospital Center, Brooklyn, USA; 2 Internal Medicine, Brooklyn VA Medical Center, Brooklyn, USA; 3 Internal Medicine, State University of New York Downstate Medical Center, Brooklyn, USA; 4 Neurology, University of Baghdad, College of Medicine, Baghdad, IRQ

**Keywords:** all neurology, endocrinology and diabetes, focal dystonia, ketosis prone diabetes, diabetes type 2

## Abstract

Diabetes is one of the most common chronic diseases affecting over 400 million patients worldwide, many of which are affected with devastating macrovascular and microvascular complications. Diabetes affects both the peripheral and the central nervous systems. One of the unusual effects of hyperglycemia is involuntary movement, termed hyperglycemia-induced involuntary movement (HIIM). Here, we present a case of a middle-aged woman with neck dystonia as the initial manifestation of type 2 diabetes. Achieving euglycemia with insulin alone resulted in complete resolution of the neck dystonia.

## Introduction

The brain relies solely on glucose for metabolism, and alterations in glucose levels with either hypo or hyperglycemia can negatively affect cerebral functions. Hypoglycemia has a more dramatic effect on cerebral function, ranging from mild reversible neuroglycopenia to irreversible damage. Hyperglycemia, on the other hand, has more subtle changes, such as alteration in regional blood flow [1,2]. Rarely, patients with hyperglycemia present with myriad abnormal movements, a condition called hyperglycemia-induced movement disorder (HIIM). Hyperglycemia-induced movement disorder (HIIM) most commonly includes hemiballismus-hemichorea but rarely can present as tremors or seizures, most of which resolve completely after normalization of blood glucose level, pointing to a metabolic etiology of the abnormal movements [3,4]. It has been suggested that high glucose levels can alter gamma-Aminobutyric acid (GABA0 metabolism leading to a reduction in seizure threshold [5]. Another possible explanation is a micro alteration in cerebral blood flow [2].

## Case presentation

A 42-year-old African-Caribbean female presented with episodes of sudden incontrollable head-turning to the left with an up-gaze lasting for about 10 seconds. The episodes recurred during the four days before admission in a random fashion with stereotypical head movement and similar duration but increased in frequency to up to five times a day. The movements were not suppressible, did not occur during sleep, and each attack resolved spontaneously.

She did not experience alteration of consciousness nor sensory or motor symptoms before, during, or after the episodes. Her past medical and surgical histories were negative and she was not taking any medications. There was no recent exposure to sick patients or any recent travel history and she had no personal or family history of seizure disorders.

Upon initial assessment, she was deemed hemodynamically stable with normal general and neurological examinations. Body mass index (BMI) was 23.1 kg/m2. Routine blood work was significant for hyperglycemia, with a postprandial glucose level of 435 mg/dL and fasting glucose level of 355 mg/dL on capillary blood glucose measurement. The metabolic panel (Table 1) showed an elevated anion gap of 16, an elevated ß-hydroxybutyrate, and a normal pH of 7.38 on venous blood gas. Hemoglobin A1c (HbA1c) was 13.7%. Anti-islet antigene (IA2) and glutamic acid decarboxylase (GAD) antibodies were undetected. During hospitalization, insulin dose was adjusted and glucose and anion gap levels decreased. 

**Table 1:** Laboratory findings of the metabolic panel Na: Sodium,  K: Potassium, Cl: Chloride, HCO3: Bicarbonate

Parameter	Day 1	Day 2	Day 3	Reference range
Na	133	136	134	136-146 mmol/L
K	4	3.7	4.2	3.5-5 mmol/L
Cl	94	104	102	98-106 mmol/L
HCO_3_	23	23	24	24-31 mmol/L
Anion gap	16	9	8	8-12
Glucose	417	298	158	70-99 mg/dL
ß-hydroxybutyrate	0.82	0.64	0.12	<0.4 mmol/L
C-peptide		0.7		1.1-4.4 ng/mL

**Figure 1:**
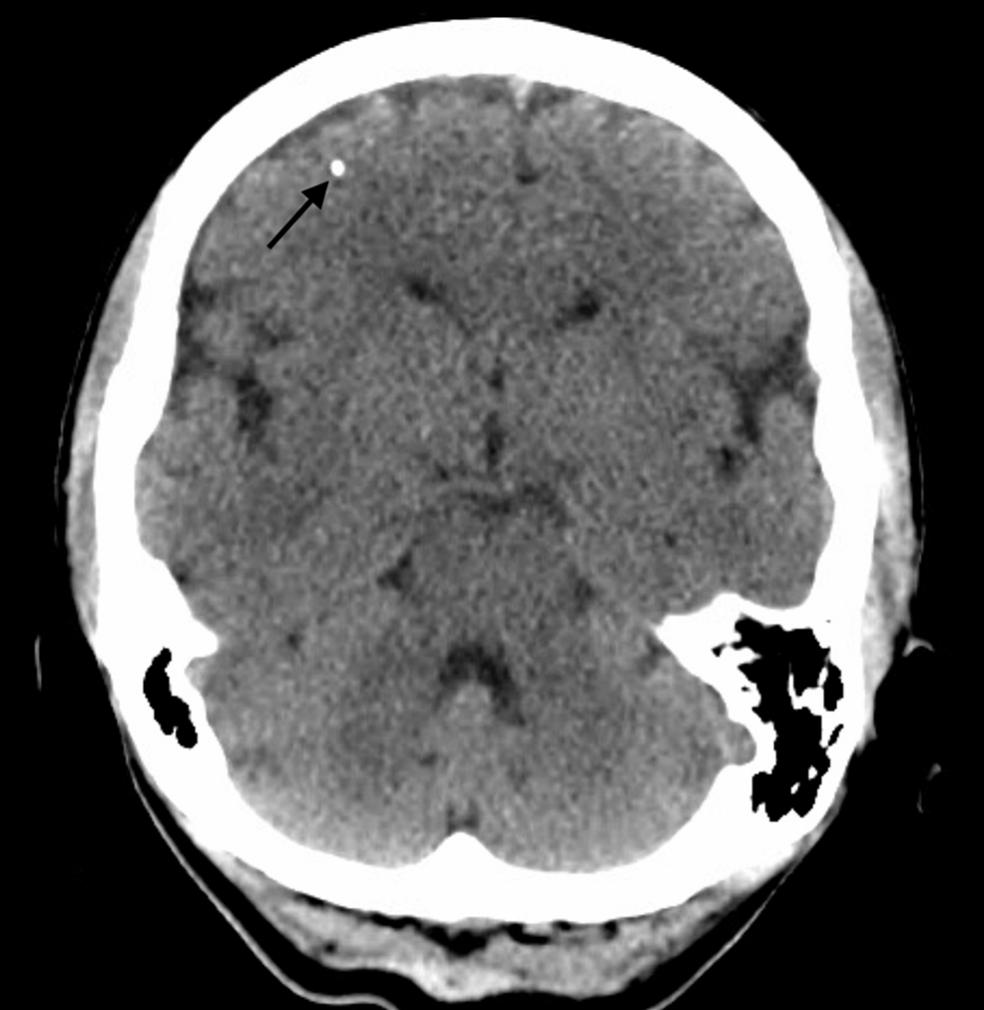
Head CT without contrast showing 1 mm calcification (arrow) in the right frontal lobe at the grey-white matter junction.

Two-hour electroencephalography (EEG) and brain MRI were normal. Brain computed tomography (CT) was unremarkable, apart from a 1 mm focus of calcification in the right frontal lobe with normal surrounding brain parenchyma (Figure 1). 

The patient did not experience any further episodes of neck dystonia during hospitalization. Follow-up at six months and 12 months after hospitalization demonstrated no recurrence of the abnormal movement. Good glycemic control was achieved with HbA1c improved to 6.1% off insulin or hypoglycemic agents, fulfilling the criteria for diabetes remission.

## Discussion

Diabetes is a disease of global concern with rising prevalence. It can affect both the peripheral and central nervous systems. Here, we report a middle-aged patient presenting with abnormal neck movement who was incidentally found to have severe hyperglycemia with ketosis and insulin deficiency, without polydipsia, polyuria weight change, or autoantibodies (anti-IA2 and GAD). This form of diabetes has been described as ketosis-prone diabetes or Flattbush diabetes. It was first described around Flatbush Avenue in Brooklyn in the 1980s in type 2 diabetes patients who presented with diabetic ketoacidosis, and then months to years later would go into remission [6]. Such patients typically present with normal BMI, very high HbA1c at presentation, high incidence of diabetic ketoacidosis, and failure of ß-cells measured by homeostatic model assessment-beta cell (HOMA-B) and homeostatic model assessment-insulin resistance (HOMA-IR) tests, with negative antibodies followed by a period of remission known as the honeymoon period. However, due to the high risk for complications, mostly retinopathy, an initial aggressive treatment regimen is warranted, justifying the use of insulin [7]. 

Hyperglycemia-induced involuntary movement (HIIM) includes hemiballismus, hemichorea, tremors, or seizures, most of which resolve completely after normalizing blood glucose levels. Dystonia is a less commonly reported manifestation, and to our knowledge, torticollis had been reported only once [3,4].

Possible mechanisms include cerebral vascular insufficiency [2], hyperglycemic or hyperosmolar insult leading to putaminal dysfunction [8], interruption of GABA transmission [5], autoimmune-mediated inflammatory process [9]. Additionally, a high HbA1c (>9%) level is associated with a higher recurrence rate of seizures which may explain the recurrence of the abnormal movements [10].

The presence of the right frontal lobe calcified focus might have served as a second-hit that potentiated the hyperglycemia to induce the involuntary movement. At the six-month follow-up, the patient was euglycemic and had not experienced any abnormal movement, ruling out the calcified focus as the sole culprit for the dystonia.

## Conclusions

Type 2 diabetes most commonly presents as polyuria and polydipsia. However, given how common diabetes is, clinicians might encounter a rare presentation of this common condition. Here, we presented such a case where diabetes presented as an involuntary movement in the form of focal dystonia of the neck. Glycemic control, in most patients, is enough to control abnormal movements. Understanding that hyperglycemia can present as abnormal movement is of paramount value to identify patients early in the downward spiral metabolic decline and salvage them with the proper treatment without further damage.
